# Association of atrial fibrillation and cancer: Analysis from two large population-based case-control studies

**DOI:** 10.1371/journal.pone.0190324

**Published:** 2018-01-11

**Authors:** Walid Saliba, Hedy S. Rennert, Naomi Gronich, Stephen B. Gruber, Gad Rennert

**Affiliations:** 1 Department of Community Medicine and Epidemiology, Lady Davis Carmel Medical Center, Haifa, Israel; 2 Ruth and Bruce Rappaport Faculty of Medicine, Technion–Israel Institute of Technology, Haifa, Israel; 3 Clalit National Cancer Control Center, Haifa, Israel; 4 USC Norris Comprehensive Cancer Center, University of Southern California, Los Angeles, California, United States of America; 5 Department of Preventive Medicine, Keck School of Medicine, University of Southern California, Los Angeles, California, United States of America; Universitatsklinikum Hamburg-Eppendorf, GERMANY

## Abstract

**Background:**

An association between atrial fibrillation (AF) and risk of cancer has been suggested in several studies, including prospective cohort studies. However, the magnitude and the temporal nature of this association remain unclear.

**Methods:**

Data from two large prospective population-based case-control studies, the Molecular Epidemiology of Colorectal Cancer (MECC, n = 8,383) and the Breast Cancer in Northern Israel Study (BCINIS, n = 11,608), were used to better understand the nature and temporality of a possible association between cancer diagnosis and AF events before and after cancer diagnosis. A case-control study approach was employed to study prior AF as a risk factor for cancer, and a cohort study approach was employed to study incident cancer as a risk factor for AF.

**Results:**

AF was associated with a significant reduced odds of cancer as reflected in the case-control approach, with an adjusted OR = 0.77 (95% CI, 0.65–0.91), while cancer was not found to be significantly associated with elevated risk of AF in the cohort approach, with an adjusted HR = 1.10 (0.98–1.23). The immediate period (90 days) after an AF event was associated with a 1.85 times increased risk of cancer, and the immediate period after the diagnosis of cancer was associated with a 3.4 fold increased risk of AF. These findings probably reflect both the effect of acute transient conditions associated with new cancer diagnosis and detection bias. Similar results were identified with colorectal and breast cancer cases.

**Conclusions:**

Atrial fibrillation of longer than 90 days duration is associated with reduced odds of new cancer diagnosis. The results of this study suggest that an association observed in prior research may be due to instances related to cancer diagnosis and detection bias rather than a causal relationship. However, there may be bias in the sampling and residual confounding that distort the associations.

## Introduction

Atrial fibrillation (AF) is a common cardiac arrhythmia that has been associated with increased risk of congestive heart failure, stroke and thromboembolism and increased mortality [1,2]. Established risk factors for AF include: older age, male sex, hypertension, diabetes mellitus, congestive heart failure, vascular diseases, smoking and alcohol abuse [3,4], and an association with cancer occurrence has also been suggested [5–9].

Cross-sectional studies showed that patients with cancer were more likely to have prevalent AF than those without cancer [5–8]. Although the temporal nature of the association cannot be determined from cross-sectional studies, it has been suggested that cancer could promote the development of AF.

A case-control study from Denmark and the Women's Health Study (WHS) showed that cancer is a significant risk factor for AF [10,11]. Notably, these studies showed that the risk of AF is increased in the first 90 days after cancer diagnosis, but not thereafter [10,11], suggesting that acute transient conditions associated with new cancer diagnosis, such as surgery and cancer related complications may contribute to the development of AF.

Studies also suggest AF as a risk factor for cancer. A significant increased risk of cancer after AF diagnosis was detected in the WHS [11] and a Danish National Registry study [12], and was much higher (3.5–5.1 fold) in the first 90 days after AF diagnosis than later (1.1–1.4 fold).

Our study was aimed at assessing the magnitude and the temporal nature of the association between cancer and AF using data from two large prospective population-based case-control studies.

## Materials and methods

### Study population

Participants in this analysis come from two large on-going population-based prospective case-control studies: the Molecular Epidemiology of Colorectal Cancer (MECC) [13], and the Breast Cancer in Northern Israel Study (BCINIS) [14]. Consecutively diagnosed patients, with colorectal and breast cancer, residing in a geographically defined area of northern Israel at time of diagnosis were eligible to participate in these studies. Breast and colon cancer free controls, matched on age, sex, ethnicity and residence (primary care clinic) were randomly selected from the same source population (living in the same area). While patients with cancer were from the entire eligible population, the sampling frame of controls was from Clalit Health Services (CHS) register of insurees residing at the same area. CHS is the largest health care provider in Israel and covers more than half of the population in Israel. Health care coverage in Israel is mandatory and is provided by four groups akin to not-for-profit health maintenance organizations. Thus, all study participants had a similar health insurance plan and similar access to health services, including prevention, and cancer screening. Recruitment to the MECC Study started March 31, 1998 and recruitment to the BCINIS started at January 1, 2000. In both studies participants provided IRB approved written informed consent at the time of enrollment and were interviewed to obtain information about their personal history of cancer, medical history, medication use, education, and health habits including alcohol consumption, smoking, and physical activity. Included in this analysis are participants who did not self-report a history of previous cancer, and who were CHS insurees (for whom computerized full prescription data, comorbidities, and AF data were available).

### The database

The electronic medical record (EMR) database of CHS includes data from multiple sources: records of primary care physicians, community specialty clinics, hospitalizations, laboratories and pharmacies [15,16]. A registry of more than 100 diagnoses of chronic diseases is compiled from these data sources. Diagnoses are captured in the registry by diagnosis-specific algorithms, employing International Classification of Diseases Ninth revision (ICD-9) code reading, text reading, laboratory test results and disease-specific drug usage. A record is kept of the data-sources and dates used to establish the diagnosis, with the earliest recorded date, from any source, considered to be the defining date of diagnosis.

### Study variables

Only newly diagnosed histologically confirmed malignancies were included in the MECC and BCINIS studies, and the date of cancer diagnosis was determined from the diagnostic pathology report. For controls the starting date was set to the date of recruitment in the study. Data on AF and date of start of diagnosis were retrieved from the CHS chronic diseases registry. Data on level of education, height, weight, cancer screening history, alcohol consumption, smoking status, and physical activity were self-reported by participants from in-person interviews. Data on medication use, and previous comorbidities such as diabetes, hypertension, vascular disease, and congestive heart failure were retrieved from the CHS chronic diseases database.

This study was reviewed and approved by the ethics committee of the Carmel Medical Center before it began.

### Study design

To explore the temporal nature of the association between AF and cancer, we used two analytic approaches within the case-control studies. A case-control study approach was employed to study prior AF (exposure) as a risk factor for cancer (outcome), and a cohort study approach was employed to study incident cancer (exposure) as a risk factor for AF (outcome).

### Statistical analysis

Continuous variables were summarized with mean ± SD, and categorical variables were presented as numbers and proportions. Comparisons of baseline characteristics between cases and controls were performed using the student *t* test for continuous variable and the chi-square test for categorical variable. Logistic regression was used in the case-control approach analysis to assess the association between exposure to AF and cancer. Exposure to AF was classified into different categories based on the time interval elapsed between AF diagnosis and cancer diagnosis that were compared to those without prior AF. Cox proportional hazard regression models were used in the cohort approach analysis. To assess whether the risk of AF differs according the time elapsed after the diagnosis of cancer, we constructed Cox models for incident AF using four separate cancer indicator variables for the period of 0–3 months, 3 months-1year, 1–3 years, and beyond 3 years of cancer diagnosis. Multivariate logistic regression and Cox proportional hazard regression models were adjusted for age, sex, smoking, alcohol consumption, physical activity, education, medications use (aspirin, statins, and anticoagulants), and comorbidities (hypertension, diabetes, congestive heart failure, and cardiovascular disease). Because BMI was missing in 1,083 (5.4%) patients, we performed a separate analysis by including BMI in the multivariate models. In addition we adjusted for HRT use in a separate analysis that was restricted to women participants in our study.

Because we included in the study only CHS insurees and excluded participants who self-reported a prior history cancer, the original matching was not conserved in all participants. Hence, we performed sensitivity analysis by including only matched sets of cases and controls. For the matched analysis we used conditional logistic regression in the case-control study analytic approach, and for the cohort study analytic approach we used Cox proportional hazard regression analysis stratified by matched sets.

All statistical analyses were performed using IBM SPSS Statistics 23.0 (IBM, New York, NY). For all analyses, P < 0.05 for the 2-tailed tests was considered to be statistically significant.

## Results

Our study group includes 19,991 CHS insurees; of them 9,264 are incident cancer cases and 10,727 controls free of cancer at time of recruitment (Table 1). The mean age of cases was 63.6 ± 13.6 years and of controls was 64.3 ± 13.6 years, and 76.4% of cases were women compared to 79.3% of controls. Table 1 displays the comparisons of baseline characteristics between cases and controls and between those with prior AF and no prior AF among cases and controls. Most of the demographic and clinical variables distributions are significantly different between cancer cases and controls. However, effect sizes are really small and therefore differences can be neglected. Individuals with prior AF are older, have higher frequency of comorbidities and are more likely to be treated with statins and antithrombotic medications (Table 1).

**Table 1:** Demographic and clinical baseline characteristics of study participants presented in cases and controls and by prior atrial fibrillation status among cases and controls (n = 19,991).

Variable	Cases and controls		Prior atrial fibrillation (AF) status among cases and controls	
	Cases	Controls	AF	no-AF
	*(n = 9264)*	*(n = 10727)*	*(n = 890)*	*(n = 19101)*
**Age**	63.6 ± 13.6	64.3 ± 13.6*	76.0 ± 9.0	63.5 ± 13.6*
**Females**	7,080 (76.4%)	8,502 (79.3%)*	586 (65.8%)	14,996 (78.5%)*
**Diabetes mellitus**	1,820 (19.6%)	2,147 (20.0%)	318 (35.7%)	3649 (19.1%)*
**Hypertension**	4,164 (44.9%)	4,868 (45.4%)	770 (86.5%)	8,262 (43.3%)*
**Congestive heart failure**	309 (3.3%)	423 (3.9%)*	247 (27.8%)	485 (2.5%)*
**Vascular disease**	1,532 (16.5%)	1,990 (18.6%)*	556 (62.5%)	2,966 (15.5%)*
**Atrial fibrillation**	352 (3.8%)	538 (5.0%)*	-	-
**CHA**_**2**_**DS**_**2**_**-VASc score**	2.45 ± 1.66	2.56 ± 1.71*	4.63 ± 1.70	2.41 ± 1.62*
**Smoking**	3,092 (33.7%)	3,973 (37.1%)*	313 (35.2%)	6,752 (35.5%)
**Alcohol consumption**	1,611 (17.6%)	2,075 (19.4%)*	158 (17.8%)	3,528 (18.6%)
**Education** (>12 years)	3,628 (39.3%)	4,605 (43.0%)*	302 (34.1%)	7,931 (41.6%)*
**Physical activity**	3,201 (34.9%)	4,544 (42.4%))	237 (26.7%)	7,508 (39.5%)*
**Body mass index (BMI)**				*
< 25 Kg/m^2^	3,228 (37.3%)	3,723 (36.3%)	269 (32.7%)	6,682 (36.9%)
25–30 Kg/m^2^	3,268 (37.7%)	3,943 (38.5%)	302 (36.7%)	6,909 (38.2%)
≥ 30 Kg/m^2^	2,168 (25.0%)	2,578 (25.2%)	251 (30.5%)	4,495 (24.9%)
**Aspirin**	2,424 (26.2%)	3,009 (28.1%)*	413 (46.4%)	5,020 (26.3%)*
**Statins**	3,196 (34.5%)	3,990 (37.2%)*	554 (62.2%)	6,632 (34.7%)*
**Anticoagulants**	298 (3.2%)	384 (3.6%)	420 (47.2%)	262 (1.4%)*

* P value <0.05

### Atrial fibrillation as a risk factor for cancer

The association between AF (exposure) and cancer (outcome) was assessed using a case-control approach analysis. A total of 19,991 participants were included in this analysis. Logistic regression models were used to estimate the OR for cancer associated with prior AF and with the different categories of AF duration, using those without prior AF as reference category. A previous history of AF was detected in 352 (3.8%) cases and 538 (5.0%) controls, and was significantly associated with lower risk of cancer, with an adjusted OR = 0.77 (95% CI, 0.65–0.91) (Table 2). When we considered the time elapsed between AF diagnosis and cancer diagnosis, we detected a nonsignificant temporary increase in the odds of cancer diagnosis in the first 90 days after AF diagnosis, with adjusted OR = 1.85 (95% CI, 0.98–3.49), while after 90 days the odds of cancer diagnosis was significantly reduced (adjusted OR = 0.73, 95% CI, 0.61–0.87). The results remained consistent after 3 years of AF diagnosis (adjusted OR = 0.70, 95% CI, 0.57–0.86) (Table 2). Further adjustment for BMI yielded similar results. The results were similar after further adjustment for HRT use among women participants in the study. Cancer odds was 2.11 (0.87–5.14) in the first 3 months of AF diagnosis, and 0.78 (0.63–0.97) with AF of longer than 3 months. We reached similar results when the associations with colorectal and breast cancer were assessed separately (S1 Table).

**Table 2:** Crude and adjusted odds ratios (ORs)* for the association of exposure to atrial fibrillation and cancer in the case-control analysis *(n = 19*,*991)*.

Exposure categories	Cancer cases and controls		Crude *OR (95% CI)*	Age adjusted *OR (95% CI)*	Fully adjusted** *OR (95% CI)*	P value
	Cases *(n = 9*,*264)*	Controls *(n = 10*,*727)*				
***AF any time before cancer***						
no-AF	8,912 (96.2%)	10,189 (95.0%)	Reference	Reference	Reference	
Yes	352 (3.8%)	538 (5.0%)	0.75 (0.65–0.86)	0.78 (0.67–0.89)	0.77 (0.65–0.91)	0.002
***Time of AF before cancer***						
no-AF	8,912 (96.2%)	10,189 (95.0%)	Reference	Reference	Reference	
≤3 months	26 (0.3%)	16 (0.1%)	1.86 (1.0–3.47)	1.93 (1.03–3.59)	1.85 (0.98–3.49)	0.058
>3 months	326 (3.5%)	522 (4.9%)	0.71 (0.62–0.82)	0.74 (0.64–0.86)	0.73 (0.61–0.87)	<0.001
***Time of AF before cancer***						
no-AF	8,912 (96.2%)	10,189 (95.0%)	Reference	Reference	Reference	
≤ 3 months	26 (0.3%)	16 (0.1%)	1.86 (1.0–3.47)	1.93 (1.03–3.59)	1.85 (0.98–3.49)	0.058
>3 months–1 year	40 (0.4%)	57 (0.5%)	0.80 (0.53–1.20)	0.83 (0.55–1.25)	0.79 (0.51–1.20)	0.265
1 year–3 years	85 (0.9%)	129 (1.2%)	0.75 (0.57–0.99)	0.78 (0.59–1.02)	0.77 (0.57–1.03)	0.075
> 3 years	201 (2.2%)	336 (3.1%)	0.68 (0.57–0.82)	0.71 (0.59–0.85)	0.70 (0.57–0.86)	0.001

*; logistic regression models were used to estimate the OR for cancer associated with prior AF and with the different categories of AF duration, using those without prior AF as reference category

**; adjusted for age, sex, hypertension, diabetes mellitus, congestive heart failure, vascular diseases, medications use (aspirin, statins, anticoagulants), physical activity, education, smoking, and alcohol consumption.

Matched analysis with conditional regression models, including 13,624 participants, showed similar results, with elevated odds of cancer in the first 90 days after AF diagnosis (adjusted OR = 3.0, 95% CI, 1.23–7.31) and decreased odds of cancer after 90 days (adjusted OR = 0.62, 95% CI, 0.50–0.77) (S2 Table).

### Cancer as a risk factor atrial fibrillation

The association between cancer (exposure) and AF (outcome) was assessed using a cohort approach analysis. Of the 19,991 participants, 19,101 were included in this analysis (890 participants with previous history of AF were excluded). Of the 8,912 patients with cancer, 588 developed AF during 58,041 person years (rate 10.1 per 1000 person-years), and of the 10,189 participants without cancer, 667 developed AF during 70,189 person years (rate 9.5 per 1000 person years) (Table 3). New onset cancer was not significantly associated with AF during the entire follow up period (adjusted HR, 1.10, 95% CI, 0.98–1.23). However, when the association was examined at different time intervals cancer was significantly associated with AF in the first 90 days following cancer diagnosis, but not beyond 90 days (adjusted HRs, 3.40 (95% CI, 2.06–5.61) and 1.02 (95% CI, 0.91–1.15), respectively) (Table 3). The results were similar after further adjustment for HRT use among women participants in the study. The risk of AF was 3.05 (1.64–5.68), and 1.01 (0.87–1.16) in the first 3 months and beyond 3 months of cancer diagnosis, respectively.

**Table 3:** Crude and adjusted hazard ratios (HRs)* for the association of exposure to cancer and new incident atrial fibrillation in the cohort analysis *(n = 19*,*101)*.

Time interval for incident AF after cancer diagnosis	Exposure		Crude *HR (95% CI) Cancer vs*. *no-cancer*	Age adjusted *HR (95% CI) Cancer vs*. *no-cancer*	Fully adjusted** *HR (95% CI) Cancer vs*. *no-cancer*	P value
	Cancer (cases) *(n = 8*,*912) Events/at risk*	Controls *(n = 10*,*189) Events/at risk*				
Any time	588/8912	667/10189	1.07 (096–1.20)	1.12 (1.0–1.25)	1.10 (0.98–1.23)	0.109
≤3 months	60/8912	21/10189	3.29 (2.0–5.40)	3.45 (2.10–5.68)	3.40 (2.06–5.61)	<0.001
>3 months	528/8755	646/10090	1.0 (0.89–1.12)	1.04 (0.93–1.17)	1.02 (0.91–1.15)	0.739
>3 month-1 year	57/8755	51/10090	1.29 (0.88–1.89)	1.37 (0.94–2.0)	1.29 (0.87–1.89)	0.199
1 year-3 years	108/8374	138/9670	0.92 (0.71–1.18)	0.98 (0.76–1.27)	0.96 (0.74–1.24)	0.743
>3 years	363/6664	457/7770	0.99 (0.86–1.13)	1.03 (0.89–1.18)	1.01 (0.88–1.16)	0.897

*; Cox proportional hazard regression models were used to estimate the HR for incident AF using separate cancer indicator variables for the period of cancer diagnosis

**; adjusted for age, sex, hypertension, diabetes mellitus, congestive heart failure, vascular diseases, medications use (aspirin, statins, anticoagulants), physical activity, education, smoking, and alcohol consumption.

The results were similar on matched analysis (S2 Table), and when the associations with colorectal and breast cancer were assessed separately (S1 Table).

## Discussion

A significant inverse association between AF and future development of malignancy was identified in our study. This finding held true in adjusted matched and unmatched analyses, and separately for breast cancer and for colorectal cancer risk.

No association was identified in a cohort of cancer cases and future development of AF. However, the immediate period (90 days) after the diagnosis of cancer was associated with an increased risk of AF. A similar immediate increased risk of AF following diagnosis of cancer was reported in a recent analysis from the Women's Health Study (WHS) [11], and a case-control study from Denmark [10]. Similar to our study, both studies showed an increased risk of AF in the first 90 days after cancer diagnosis, but not beyond 90 days [10,11]. The lack of association beyond 90 days between newly diagnosed cancers and incident AF in our study, suggests that the observed strong association in the first 90 days might be related either to detection bias or to acute transient conditions associated with new cancer diagnosis, such as adverse effect of invasive diagnostic procedures and treatment (medical and surgical) which are usually performed close to the time of cancer diagnosis [9–11,17]. Emotional stress, and pain associated with newly diagnosed cancer may also precipitate AF [9]. Furthermore, at the time of diagnosis cancer may present with acute complication, such as acute infections, inflammation dehydration, bleeding, thrombosis, anemia, and constipation/ileus which as well may precipitate AF [9,10,12,18,19].

The immediate period (90 days) after an AF diagnosis was associated with an increased risk of cancer. However, the small sample size for patients with AF before ≤3 months of cancer diagnosis may be problematic as evidenced by the large 95% CI. A similar immediate increased risk of cancer following diagnosis of AF was reported in the WHS [11], and a cohort study from Denmark [12]. During this short period cancer is likely to exist before AF, suggesting reversal causality [12]. After the first 90 days of AF diagnosis, the risk estimates decreased significantly in the WHS (HR from 3.54 to 1.39) [11], and the decrease was even more dramatic in the Danish cohort study (SIR from 5.11 to 1.13) [12]. In our study however, the risk of cancer after the first 90 days of AF diagnosis was lower in patients with AF compared to patients without AF (adjusted OR, 0.73, 95% CI, 0.61–0.87). This discrepancy between the results of these studies and our study may be in part explained by residual confounding and ascertainment of AF and cancer diagnosis. The Danish study lacks of adjustment for potential confounders and compared the observed rates of cancer in the study AF patients with the expected rates in the general population [12].

The decreased risk of cancer associated with AF in our study may be mediated by any number of possibilities, and some of the potential relationships are complex. For example, long-term anticoagulation is fundamental to the management of AF, and anticoagulation is related to both the incidence and diagnosis of colorectal cancer. Unlike the United States, colorectal cancer screening in Israel is driven largely by fecal occult blood testing or, more recently, fecal immunochemical testing [20–22]. Anticoagulation doubles the positivity rate of fecal occult blood testing [20]. In addition, aspirin and screening are both strongly and independently related to reduced risk of colorectal cancer [23]. We adjusted for aspirin and anticoagulation use, and this did not influence the inverse relationship we observed between AF and colorectal cancer.

Similarly, the relationship of hormone replacement therapy (HRT) and cancer is complex. HRT decreases risk of colorectal cancer [24], but increases the risk of breast cancer and has a complex relationship with coronary heart disease [25]. HRT is also associated with increased risk of AF [26]. In our study, the long-term inverse relationship between a prior history of AF and cancer was nearly identical for colorectal cancer and breast cancer, suggesting that the HRT is unlikely to mediate any relationships between AF and cancer risk. In addition, we reached similar results when we adjusted for HRT use among women participants in our study.

Atrial natriuretic peptide (ANP) has been shown to inhibit tumor growth in vitro and in vivo studies [27, 28]. ANP belongs to a family of cardiac and vascular-derived peptide hormones that plays a crucial role in cardiovascular homeostasis through blood pressure and volume regulation [29,30]. AF is an independent determinant of ANP, yet the mechanism of increased ANP in AF remains unclear [31]. ANP appears to have anti-proliferative effect that has been extensively demonstrated in various forms of human cancer including pancreatic carcinoma, breast carcinoma, small cell lung carcinoma, and colorectal carcinoma [27, 28]. Serafino et al have demonstrated that the inhibition of tumor cell proliferation by ANP is mediated by a concomitant effect on the intracellular acidity and the Wnt/β-catenin signaling [32]. In addition, the two years relapse-free survival after curative surgery for lung cancer was significantly greater in ANP-treated patients than in control patients (91% vs. 67%, P = 0.018) [33]. The authors found that ANP inhibited the adhesion of cancer cells to pulmonary arterial and micro-vascular endothelial cells by suppressing the E-selectin expression that is promoted by inflammation [33].

The higher frequency of females (77.9%) in our case-control studies stems from the breast cancer study that included females. The proportion of women in the colorectal cancer case-control study was 47.5%. Furthermore the results were consistent when breast and colorectal cancer were analyzed separately. Thus, the difference in sex frequency in our study is unlikely to introduce bias. Unfortunately we don’t have data on other cancers as our analysis was confined to colorectal and breast cancer using data from two large population-based case-control studies available to us. The external validity and the extension of our conclusions to other cancers remains a matter or reasoning. The robustness of our findings when colorectal and breast cancer were analyzed separately suggests that this may be true for other cancers. However, future studies are needed to solve this issue. Another limitation of our study is that the diagnosis of atrial fibrillation was retrieved from the CHS database by means of ICD-9 code reading. However, a previous study from the same database showed that the prevalence and incidence of atrial fibrillation were comparable to those reported in Europe and North America [34]. Naturally we cannot exclude that some non-differential misclassification has occurred, however such misclassification is expected to bias the results toward the null. Our study is observational in nature. As such, it cannot prove cause-effect relationships. Residual confounding from unmeasured and unknown covariates remains a concern. Regardless, the issue of confounding should not undermine the importance of our findings. If present, confounding suggests that AF and cancer may share opposite risk factors, meaning that some risk factors for AF may have beneficial effect in preventing future cancer. Identification of these factors in future studies may have important public health implications for cancer prevention.

## Conclusions

AF is associated with short-term increased risk of diagnosis of cancer, but decreased long-term risk of future cancer.

## Supporting information

S1 Data Original study data. (DAT)

S1 Table Association between atrial fibrillation (AF) and cancer presented separately for colorectal and breast cancer in the cohort and the case control analysis. (PDF)

S2 Table Matched analysis for the association between atrial fibrillation (AF) and cancer in the cohort and the case control analysis. (PDF)
