# Supplementary material for: Association of atrial fibrillation and cancer: Analysis from two large population-based case-control studies
Source: PLoS One. 2018 Jan 11;13(1):e0190324. doi: 10.1371/journal.pone.0190324 (PMC5764256; doi:10.1371/journal.pone.0190324)
Supplement: S1 Table — (PDF) [file pone.0190324.s002.pdf]

S1 Table. Association between atrial fibrillation (AF) and cancer presented separately for colorectal and breast cancer in the cohort and the case control analysis

**The case control analysis*: association between AF (exposure) and cancer (outcome)**				
	**Colorectal cancer** *(n=8,383)*		**Breast cancer** *(n=11,608)*	
Time of AF before cancer	*Adjusted OR (95% CI)*	*P value*	*Adjusted OR (95% CI)*	*P value*
no-AF	Reference		Reference	
≤3 months	2.01 (0.97-4.18)	0.061	1.72 (0.45-6.57)	0.425
>3 months	0.72 (0.57-0.90)	0.004	0.75 (0.58-0.98)	0.038
>3 years	0.69 (0.53-0.91)	0.009	0.73 (0.53-0.99)	0.044
**The cohort analysis**: association between cancer (exposure) and AF (outcome)**				
	**Colorectal cancer** *(n=7,881)*		**Breast cancer** *(n=11,220)*	
Time interval for incident AF after cancer diagnosis	*Adjusted HR (95% CI) Cancer vs. no-cancer*	*P value*	*Adjusted HR (95% CI) Cancer vs. no-cancer*	*P value*
≤3 months	3.89 (1.97-7.69)	<0.001	2.74 (1.29-5.81)	0.009
>3 months	0.92 (0.79-1.07)	0.266	1.22 (1.01-1.46)	0.036
>3 years	0.90 (0.75-1.08)	0.258	1.24 (0.99-1.56)	0.060

*; logistic regression models were used in the case control analysis to estimate the OR for cancer associated with prior AF and with the different categories of AF duration, using those without prior AF as reference category

**; Cox proportional hazard regression models were used in the cohort analysis to estimate the HR for incident AF using separate cancer indicator variables for the period of cancer diagnosis
