# Supplementary material for: Association of atrial fibrillation and cancer: Analysis from two large population-based case-control studies
Source: PLoS One. 2018 Jan 11;13(1):e0190324. doi: 10.1371/journal.pone.0190324 (PMC5764256; doi:10.1371/journal.pone.0190324)
Supplement: S2 Table — (PDF) [file pone.0190324.s003.pdf]

S2 Table. Matched analysis for the association between atrial fibrillation (AF) and cancer in the cohort and the case control analysis

**The case control design*: association between AF (exposure) and cancer (outcome)** *(n=13,624)*		
Time of AF before cancer	***Adjusted OR (95% CI)***	***P value***
no-AF	Reference	
≤3 months	3.0 (1.23-7.31)	0.015
>3 months	0.62 (0.50-0.77)	<0.001
>3 years	0.58 (0.45-0.75)	<0.001
**The cohort design**: association between cancer (exposure) and AF (outcome)** *(n=12,490)*		
Time interval for incident AF after cancer diagnosis	***Adjusted HR (95% CI)*** *Cancer vs. no-cancer*	***P value***
≤3 months	8.16 (2.41-27.56)	0.001
>3 months	0.98 (0.81-1.20)	0.863
>3 years	0.89 (0.70-1.15)	0.379

*; conditional logistic regression models were used in the case control analysis to estimate the OR for cancer associated with prior AF and with the different categories of AF duration, using those without prior AF as reference category

**; Cox proportional hazard regression models stratified by matched sets were used in the cohort analysis to estimate the HR for incident AF using separate cancer indicator variables for the period of cancer diagnosis
